# A Deep Learning Model for Correlation Analysis between Electroencephalography Signal and Speech Stimuli

**DOI:** 10.3390/s23198039

**Published:** 2023-09-23

**Authors:** Michele Alessandrini, Laura Falaschetti, Giorgio Biagetti, Paolo Crippa, Simona Luzzi, Claudio Turchetti

**Affiliations:** 1 Department of Information Engineering, Università Politecnica delle Marche, Via Brecce Bianche 12, I-60131 Ancona, Italy; m.alessandrini@univpm.it (M.A.); l.falaschetti@univpm.it (L.F.); p.crippa@univpm.it (P.C.); c.turchetti@univpm.it (C.T.); 2 Neurology Clinic, Department of Experimental and Clinical Medicine, Università Politecnica delle Marche, Torrette, I-60126 Ancona, Italy; s.luzzi@staff.univpm.it

**Keywords:** canonical correlation analysis (CCA), deep correlation analysis (DCCA), deep learning, electroencephalography (EEG), multilayer perceptron (MLP), speech–EEG analysis

## Abstract

In recent years, the use of electroencephalography (EEG) has grown as a tool for diagnostic and brain function monitoring, being a simple and non-invasive method compared with other procedures like histological sampling. Typically, in order to extract functional brain responses from EEG signals, prolonged and repeated stimuli are needed because of the artifacts generated in recordings which adversely impact the stimulus-response analysis. To mitigate the artifact effect, correlation analysis (CA) methods are applied in the literature, where the predominant approaches focus on enhancing stimulus-response correlations through the use of linear analysis methods like canonical correlation analysis (CCA). This paper introduces a novel CA framework based on a neural network with a loss function specifically designed to maximize correlation between EEG and speech stimuli. Compared with other deep learning CA approaches (DCCAs) in the literature, this framework introduces a single multilayer perceptron (MLP) network instead of two networks for each stimulus. To validate the proposed approach, a comparison with linear CCA (LCCA) and DCCA was performed, using a dataset containing the EEG traces of subjects listening to speech stimuli. The experimental results show that the proposed method improves the overall Pearson correlation by 10.56% compared with the state-of-the-art DCCA method.

## 1. Introduction

The exploration of the human brain has gained the attention of both the scientific and engineering communities. One prevalent approach in this analysis involves measuring the evoked brain response to specific stimuli and subsequently establishing a correlation between them. Electroencephalography (EEG) is a simple, non-invasive method for collecting brain signals with adequate temporal resolution suitable for auditory analysis. However, due to the scalp-level measurements involved in EEG recordings, they are notably affected by noise [1].

To address this noise issue, a widely employed method is the analysis of data using the event-related potential (ERP) technique [2]. This involves averaging brain potentials from numerous repetitions of input stimuli under a specific condition, effectively eliminating out-of-phase components while retaining stimulus-related components. Nonetheless, this approach has limitations as it primarily applies to isolated short stimuli that must be repetitively presented, making it less suitable for analyzing natural continuous stimuli such as speech and music.

A notable advancement in this field is represented by the temporal response function (TRF), proposed by Lalor et al. [3,4]. The linear TRF model characterizes the connection between a stimulus and its response as a linear time-invariant system. The TRF is a model that describes how a sudden change in an input feature corresponds to a change in the EEG data, forming a stimulus-response relationship. Unlike characterizing the response to repetitions of the same stimuli, as in the ERP method, TRF allows for the generation of continuous predictions about EEG responses. Significantly, it has been shown that the stimulus-response models can be derived not only from EEG responses to artificial sound stimuli [3,5] but also from EEG responses to naturalistic speech [4].

In recent auditory–EEG analyses, canonical correlation analysis (CCA) has demonstrated superiority over forward and backward TRF models [6,7]. CCA is a robust linear technique that projects two signals into a domain that maximizes their correlation [8,9]. This method achieves this by identifying a linear transformations for each signal, minimizing the irrelevant variability with respect to the other signal. Successfully applied in auditory–EEG analysis through a combination of linear transforms and convolutions [6,10], CCA still relies on linear assumptions despite its efficacy.

In each subject’s analysis, the auditory signal is represented through two distinct “views”: the stimulus view and the response view. The stimulus view captures the audio signal by employing a temporal envelope, while the response view represents the same signal through the brain responses recorded as EEG data. The concept of feature learning with multi-view data has been previously explored using linear canonical correlation analysis (LCCA) [11], as well as its nonlinear extension known as kernel CCA (KCCA) [12,13].

In the recent decade, with the growth of deep learning techniques also in the field of brain activity decoding [14,15,16], an extension of the linear-transformation-based CCA analysis to deep-transformation-learning-based CCA was developed, first proposed by Andrew et al. [17]. Unlike the linear/kernel CCA methods, DCCA employs a deep neural network (DNN) for feature mapping instead of using a linear/kernel function.

In this paper, we propose a CA framework based on deep learning that uses the CCA as a pre-processing step to reduce the data dimensionality, and then, once extracted, the most significant features through the Karhunen–Loève transform (DKLT); these features are provided as input of a neural network optimizing the correlation between the two data views, specifically in this case, the correlation between EEG and speech stimuli. Particularly, a comparison with the LCCA and the state-of-the-art DCCA proposed by Katthi et al. [18,19] was conducted to validate the proposed approach.

The rest of the paper is organized as follows. Section 2 describes the dataset adopted in the experiments and the correlation analysis methods implemented. In Section 3, the experimental results are presented, while in Section 4, a discussion about the results and their potential applications is provided. Finally, Section 5 draws the conclusions of this work.

## 2. Materials and Methods

### 2.1. Dataset

The Speech-EEG dataset is an open source dataset available at [20]. This dataset derives from a study conducted in [21] demonstrating that the EEG reflects semantic processing of continuous natural speech. The dataset provides five subsets with different characteristics: Cocktail Party Dataset, N400 Dataset, Natural Speech - Reverse Dataset, Natural Speech Dataset, and Speech in Noise Dataset. The dataset chosen for the experimentation is the Natural Speech Dataset, which contains the following:

- EEG traces of 19 subjects engaged in 20 trials of an experiment that involved listening to a single audiobook;
- Twenty stimuli files containing the audio of a male speaker reading snippets of a novel and the associated envelope.

For all experiments, 128-channel EEG data (plus two mastoid channels) were acquired at a rate of 512 Hz using an ActiveTwo system (BioSemi). Several offline preprocessing steps were already applied to the provided dataset: the data were band-pass filtered between 1 and 8 Hz, downsampled to 128 Hz, and re-referenced to the average of the mastoid channels.

This dataset was chosen as it has been used in the literature in the application of both linear CCA [6] and deep CCA [18,19] models.

### 2.2. Proposed Method

CCA is a standard statistical technique for finding linear projections of two random vectors that are maximally correlated [17]. Instead, in the proposed method, the linear CCA is used as a dimensionality reduction technique, and the correlation of the two data views are simultaneously computed by training the neural network, specifically a multilayer perceptron (MLP), instead of using two distinct networks for each view or stimulus. Additionally, a feature extraction step was performed through the DKLT in order to provide as input of a neural network the most significant features. This model aims to enhance intra-subject stimulus-response correlations and to reduce EEG artifacts without removing the components associated with the stimulus.

**Figure 1:**
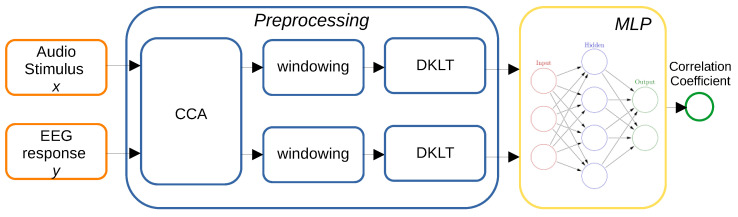
Flowchart of the proposed method.

The proposed framework is depicted in Figure 1. Input and output are first transformed through CCA (Section 2.2.2) and then individually split in segments trough rectangular windowing (Section 2.2.3). The DKLT is finally applied to the resulting set of realizations, for both inputs and outputs (Section 2.2.4). The resulting data represent the inputs and outputs for the neural network (Section 2.2.5).

#### 2.2.1. Data Preprocessing

The proposed method does not operate on the time-based original signals but rather on the spectral transformation of inputs and outputs, via a suitable algorithm, such that audio and EEG data are expressed as a set of static features according to an optimal basis.

The algorithm that has been used, together with the other preprocessing operations, will be discussed in the following subsections.

#### 2.2.2. CCA

In general, datasets composed of EEG responses to given stimuli have different properties for inputs and outputs. Input stimuli (audio signals in our case) usually have low dimensionality but a higher sampling frequency, while outputs (EEG recordings) have lower frequency but a significant number of components (EEG tracks). In order to compute the correlation between the two components (namely the Pearson correlation coefficient), it is desirable to have both the input and output as monodimensional vectors of the same size.

For this reason, several processing steps are performed on the data to be able to compute the input–output correlation. For the input audio signals, it is convenient to extract the envelope of the audio, instead of working with full-time-based audio; this has been shown to better model the dependency between auditory input and EEG output [18,21,22,23,24]. Indeed, the dataset used in this work directly includes the audio envelope as input, rather than the full audio. A further advantage is that the envelope can be computed at a lower sampling frequency, matching the EEG signal frequency.

For the EEG data, we chose to reduce the data dimensionality by computing the linear CCA between the audio envelope and the EEG and keeping only one component. This results in a decomposition for both input and outputs, where the two data are both mono-dimensional and are maximally correlated. In turn, this allows us to have a starting input and output with a better correlation with respect to the original data, where the high levels of noise and the inherent complexity of cerebral activity do not allow for sensible correlations between individual EEG tracks and input audio to be identified.

#### 2.2.3. Data Windowing

Since input and output data in this dataset, as is common in similar datasets, consist of a limited number of subjects, with associated data of varying size, a division in (overlapping) windows of fixed sizes along the time axis has been performed. This has the advantage of having a bigger number of realizations for the neural network, each one of fixed size. Indeed data windowing is normally performed in computations involving machine learning and neural networks in particular [25,26,27,28].

With *w* being the window size, measured in samples, and *o* being the number of overlapping samples, the *n*-th data window consists of the samples in the range

(1) $$\left[\;(w-o)\;n\;,\;(w-o)\;n+w-1\;\right]$$

of the original signal.

All the data windows from all the subjects are finally concatenated to obtain a data matrix of size N×w, with *N* being the total number of data windows.

It can be seen that *w* and *o* are important hyper-parameters for the proposed algorithm; the chosen values are listed in Section 3.

#### 2.2.4. DKLT

An important step in the proposed algorithm, as anticipated, is using the spectral representation of input and output data. For this, the discrete Karhunen–Loève transform (DKLT) has been used in order to separate the time-dependent components of data from their fixed features [25,26].

It is also possible, and indeed desirable, to truncate the obtained features to their principal components via a principal component analysis (PCA). In this way, the data complexity can be reduced, and often, the most relevant signal information are isolated from noise and other artifacts not conveying useful information.

If *X* is the original matrix, the method consists of using the singular value decomposition (SVD):

(2) $$X=USV^{T}$$

where *S* represents a diagonal matrix containing the singular values, and *U* and *V* are the singular vectors. Given that, a new matrix can be computed:

(3) $$X^{*}=XV$$

expressing *X* in terms of the *V* vector base.

For the PCA, it is then a matter of keeping a limited number of singular values and associated vectors of *V*. For the nature of SVD, this consists of truncating matrices to the first *p* rows and columns, with p<w, consisting of the most significant components. In this way, with $X^{*}$ being smaller than *X*, a complexity reduction is also achieved, and in turn, this can be shown to lead to better final results. The criterion for the choice of *p* is shown in Section 3.

#### 2.2.5. Neural Network

A multilayer perceptron is a type of artificial neural network that is widely used in machine learning and deep learning for various tasks, including classification, regression, and pattern recognition. It is a fundamental architecture in the field of neural networks. An MLP consists of three or more layers of interconnected nodes, or neurons, organized in a hierarchical manner:

- Input Layer: this layer receives the raw input data. Each neuron in the input layer corresponds to a feature of the input data.
- Hidden Layers: These are one or more layers that come between the input and output layers. Each neuron in a hidden layer takes input from the neurons in the previous layer and applies a linear combination of the inputs followed by a non-linear activation function. The purpose of these hidden layers is to learn complex patterns and relationships within the data.
- Output Layer: This layer produces the final output of the network. The number of neurons in the output layer depends on the specific task.

**Figure 2:**
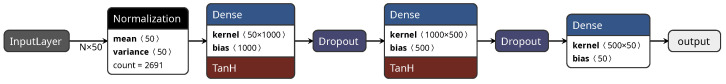
Neural network architecture.

MLP is the neural network used in this work and the implemented architecture is depicted in Figure 2: it is composed of three fully-connected layers (Dense), the first one matching the dimension of input data and the last one matching the dimension of output data, plus an additional hidden layer.

The dimension of input data is N×50, where *N* is the size of the data batch (computed automatically by the network), and 50 is the number of components retained in the PCA, as listed together with the other parameters in Section 3.

A problem arising in the training of a neural network is overfitting, that is adapting the network parameters too closely to the training data, with poor results when applied to validation and testing data, not previously seen by the network. Many strategies exist to mitigate this problem. A simple and effective one is using Dropout layers, which discard a random portion of the data at different parts of the processing chain, thus avoiding fitting the network too strictly on the same data at every step.

#### 2.2.6. Loss Function

Global optimization of the neural network relies on the use of a loss function, which drives the network training towards the optimal values of its parameters.

The choice of loss function is usually determined by the purpose of the network. As an example, for a network performing a multi-category classification, a suitable loss function is the sparse categorical crossentropy function [25,26]. In more general cases, like regression of a non-linear function, more generic loss functions can be useful, for example the mean squared error (MSE).

In this case, the purpose of the work is finding the maximally correlated components of outputs with respect to inputs, so a custom loss function has been defined with the aim of minimizing the difference between the input–output correlation of true and predicted data. Specifically, the loss function is

(4) $$J\left(W\right)=\left|\rho \left(x,\hat{y}\right)-\rho \left(x,y\right)\right|$$

where *W* is the set of network parameters; *x* is the network input; *y* and $\hat{y}$ are the true and predicted outputs, respectively, and ρ(·,·) is the Pearson correlation coefficient.

## 3. Experimental Results

The neural network was developed with TensorFlow v. 2.12.0 and Keras v. 2.12.0 on a computer with an Intel Core i7-6800K CPU, 32 GiB of RAM, and a GeForce GTX 1080 GPU.

To test the effectiveness of the network on data different from the ones used for training, for every subject, a cross-testing strategy was adopted: for every input–output pair, a different train and testing session was performed by leaving the given pair out of training and using it as testing, with the rest of the data used for training and validation. Finally, the average results were computed for the given subject.

**Figure 3:**
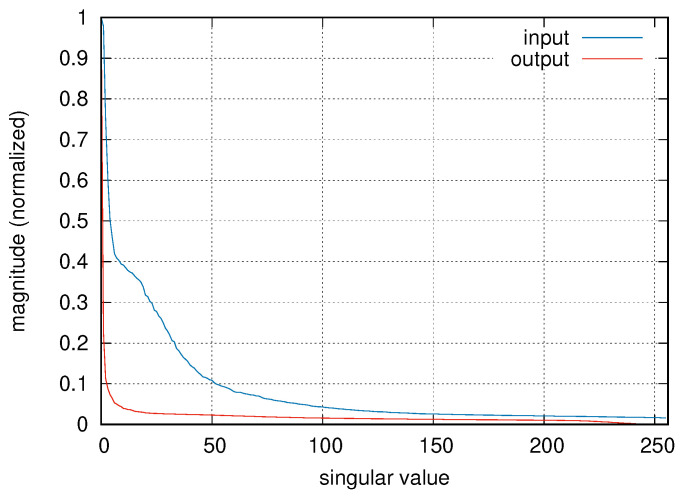
Singular values for input and output data of a sample subject.

For data preprocessing, input and output data were split in windows of 256 samples and a 50% overlap. When performing the DKLT, 50 components were retained for the PCA; this value was empirically determined according to the relative magnitude of the singular values for the given cases (an example is shown in Figure 3).

Table 1 shows the results for all the subjects in the dataset, in terms of Pearson correlation coefficient of input–output data. The values in the second column refer to the LCCA method that was computed on the two original data views, that is, the envelope of the original wave file and the EEG for each subjects. The Pearson correlation coefficients reported in the third column were computed using as views the original audio envelope and the output (predicted EEG) of the neural network.

As a matter of comparison, Table 2 compares the results with the ones from [18], the same eight subjects (1, 6, 7, 10, 11, 13, 17, 19) that were used in the other work [29]. The average of the Pearson correlation values for the aforementioned subjects was also computed.

## 4. Discussion

In this paper, a method to maximize the correlation between audio stimuli and resulting EEG signals was developed using a neural network on a preprocessed representation of input and output.

Some experiments were performed to validate this approach, using a publicly available dataset based on EEG traces acquired while different subjects were stimulated listening to continuous natural speech.

In the first experiment, we applied the proposed method to the data in order to test the effectiveness of our approach, and a comparison of the numerical results with the linear CCA method was conducted. From Table 1, it can be seen that a sensible increase in the correlation coefficient is achieved by the neural network with respect to the application of the LCCA.

In the second experiment, a deeper investigation was conducted by comparing our approach with another deep-learning based method, the DCCA proposed by [18]. This analysis, as reported in Table 2, shows that the results are comparable with an improvement achieved in five subjects out of eight.

With respect to the related works in this field [17,18,19], the first novelty consists in the use of a single neural network to obtain the maximum correlation between the EEG signals and the audio stimuli. This leads to a reduction in the computational complexity: the two neural networks used in the DCCA model by [18] have two hidden layer architectures, for each view, with 2038 and 1608 units for the first and second layers, while in the proposed method, a single neural network with two hidden layers of 1000 and 550 units, respectively, was implemented.

An additional distinction lies in how the CCA is employed: not as a linear technique for projecting two signals into a domain that maximizes their correlation but rather as a method for reducing dimensionality.

Furthermore, the approach involves transforming the EEG and speech signals from their original time-series format into a spectral representation using the established DKLT technique [25,26]. This separation effectively distinguishes the time-dependent elements from the fixed features within the signal. An advantage of this approach is the ability to truncate the number of these features to their principal components. This serves to simplify the problem’s complexity, extracting relevant information while minimizing the impact of noise and components with limited informative value.

A limitation of this approach is that the parameters of the algorithm (window size, overlap, and number of principal components of the DKLT) must be manually tuned according to the nature of the signals. Currently, the parameters have been computed for speech inputs; therefore, an analogue procedure is needed to extend its use to other datasets, such as musical stimuli.

## 5. Conclusions

In this work, a deep learning model for correlation analysis between EEG signal and speech stimuli has been proposed. This deep learning approach maximizes the correlation, using a single MLP network with a custom loss function and a pre-processing step that reduces the data dimensionality. Experimental results show the superiority of the proposed method with respect to the standard LCCA and the state-of-the-art DCCA method. Possible future work will focus on extending this approach to deep multiway CCA in order to maximize the inter-subject correlation.

## Figures and Tables

**Table 1:** Pearson correlation values for LCCA and the proposed method on the Speech-EEG dataset.

Subject	LCCA	Proposed Method
1	0.020	0.338
2	0.032	0.223
3	0.027	0.218
4	0.034	0.386
5	0.031	0.432
6	0.046	0.436
7	0.026	0.207
8	0.029	0.352
9	0.030	0.318
10	0.049	0.432
11	0.032	0.332
12	0.038	0.398
13	0.031	0.222
14	0.038	0.441
15	0.047	0.306
16	0.032	0.197
17	0.038	0.352
18	0.039	0.235
19	0.037	0.365
Overall	0.035	0.326

**Table 2:** Comparison of the Pearson correlation values for LCCA, DCCA [18], and the proposed method on the Speech-EEG Dataset using the same eight subjects (1, 6, 7, 10, 11, 13, 17, 19) chosen in [18,29]. Best results are displayed in bold.

	LCCA	DCCA [18]	Proposed Method
	0.008	0.275	**0.338**
	0.045	0.316	**0.436**
	0.020	**0.213**	0.207
	0.040	0.403	**0.432**
	0.020	**0.338**	0.332
	0.008	**0.354**	0.222
	0.019	0.292	**0.352**
	0.033	0.232	**0.365**
Overall	0.024	0.303	**0.335**

## Data Availability

This work used data publicly available from [20,21].
